# Digital refocusing and extended depth of field reconstruction in Fourier ptychographic microscopy

**DOI:** 10.1364/BOE.11.000215

**Published:** 2019-12-11

**Authors:** Remy Claveau, Petru Manescu, Muna Elmi, Vijay Pawar, Michael Shaw, Delmiro Fernandez-Reyes

**Affiliations:** 1 Department of Computer Science, Faculty of Engineering Sciences, University College London, London, WC1E 6BT, United Kingdom; 2 Biometrology Group, National Physical Laboratory, Teddington, TW11 OLW, United Kingdom; 3 Department of Paediatrics, College of Medicine of University of Ibadan, Ibadan, Nigeria; 4 mike.shaw@ucl.ac.uk; 5 delmiro.fernandez-reyes@ucl.ac.uk

## Abstract

Fourier ptychography microscopy (FPM) is a recently developed microscopic imaging method that allows the recovery of a high-resolution complex image by combining a sequence of bright and darkfield images acquired under inclined illumination. The capacity of FPM for high resolution imaging at low magnification makes it particularly attractive for applications in digital pathology which require imaging of large specimens such as tissue sections and blood films. To date most applications of FPM have been limited to imaging thin samples, simplifying both image reconstruction and analysis. In this work we show that, for samples of intermediate thickness (defined here as less than the depth of field of a raw captured image), numerical propagation of the reconstructed complex field allows effective digital refocusing of FPM images. The results are validated by comparison against images obtained with an equivalent high numerical aperture objective lens. We find that post reconstruction refocusing (PRR) yields images comparable in quality to adding a defocus term to the pupil function within the reconstruction algorithm, while reducing computing time by several orders of magnitude. We apply PRR to visualize FPM images of Giemsa-stained peripheral blood films and present a novel image processing pipeline to construct an effective extended depth of field image which optimally displays the 3D sample structure in a 2D image. We also show how digital refocusing allows effective correction of the chromatic focus shifts inherent to the low magnification objective lenses used in FPM setups, improving the overall quality of color FPM images.

## 1. Introduction

Fourier ptychography microscopy (FPM) is a computational imaging technique in which a series of low resolution brightfield and darkfield images, captured by illuminating the sample at different angles, are combined through an iterative phase retrieval algorithm to generate a single high resolution complex image with a large field of view (FoV) [1]. By relaxing the traditional trade-off between numerical aperture (NA) and magnification, FPM greatly increases the information gathering power of a light microscope and has significant potential for applications requiring both a large FoV and high spatial resolution such as imaging fixed and live cells [2–4] and tissue sections [5,6] as well as non-biological samples [7]. Conventionally the sample is assumed to be sufficiently thin that illumination with an oblique plane wave shifts the captured portion of its frequency spectrum. Combining the information contained within a series of such shifted passbands results in an image with an extended spatial frequency support, corresponding to an increase in the effective NA of the microscope from *NA*_obj_ to *NA*_syn_* = NA*_obj_ + *NA*_illu_ [8], where *NA*_illu_ is the NA corresponding to the largest illumination angle. Conventionally the depth of field (DoF) of a microscope with incoherent illumination [9] is given by

(1) $$DoF=\frac{\lambda n}{NA^{2}}+\frac{n}{M\cdot NA}e,$$

where *n* is the refractive index of the immersion medium, *M* is the magnification and *e* the camera pixel size. As Inoué and Oldenbourg note in [9] ‘in general the DoF increases, up to a factor of two as the [spatial] coherence of the source increases’. As FPM relies on fully or partially spatially coherent illumination, we expect the DoF to be somewhat larger than this. For illustration, with typical FPM parameters of *NA*_obj_ = 0.3 and *NA*_illu_ = 0.6, *λ* = 0.632 µm, *M* = 10 and *e* = 4.25 µm Eq. (1) gives the DoF as 8.3 µm for a raw image and 1.2 µm for a reconstructed image. As a result, FPM images of even relatively thin samples typically contain out of focus information and it is not possible to visualise the full thickness of the sample in a single reconstructed 2D FPM image.

Recently, several reconstruction schemes have been proposed for 3D FPM imaging. Notably, Horstmeyer et al. [10] developed a method based on diffraction tomography to reconstruct the 3D refractive index distribution of weakly scattering samples obeying the first Born approximation [11]. Tian and Waller [12] extended the basic 2D FPM reconstruction method using a coherent multi-slice model to reconstruct the complex transmittance of the object and further developed this approach to recover the 3D refractive index distribution of multiple-scattering samples including *C. elegans* embryos and an entire adult *C. elegans* specimen [13]. However, the wider application of these methods, particularly for FPM imaging of clinically relevant samples such as blood films and tissues, remains limited by their assumptions about the scattering properties of the sample and the significant increase in computational complexity. Specifically, the first Born approximation is valid for the case *kt*δ*n*/2 << 1 [10], where *k* is the wavenumber of the illumination, *t* is the sample thickness and **δ*n* is the difference between the refractive index of the sample and the immersion medium. At a wavelength of **λ** = 0.63 µm and a typical biological sample refractive index of 1.40 this requires 2*t* << 1 µm for dry samples (such as an air-dried blood film) and 0.5*t* << 1 µm for samples with oil immersion (n ∼ 1.5). These conditions are rarely met as the typical thickness of many biological samples from cell monolayers to blood films and tissue sections often exceeds 3-4 µm. In principle multi-slice methods are suitable for a wider class of 3D samples, however this comes at the expense of a very significant increase in computational load and complexity. For example, using the method described in [13], reconstruction of a 1200 × 1200 × 120 voxel volume took more than 20 hours using a high specification computer.

In this article we describe an approach for processing FPM images to allow visualization and analysis of 3D samples of intermediate thickness, which we define here as objects of thickness less than the DoF of a raw FPM image. Such samples, including tissue sections, blood films and cell monolayers, constitute a significant proportion of the sample types which would potentially benefit from FPM techniques. Reliable analysis of biomedical samples requires images within which all the relevant features are focused and resolved. This is particularly critical in the case of clinical samples for which diagnostic decisions are based on visualization of (often subtle) structural details, often in the presence of confounding artefacts due to contamination and sample preparation. Specifically, we show how digital refocusing by direct numerical propagation of the recovered complex image allows accurate visualization and analysis of the 3D structure of the sample. In contrast to previous work [1] in which digital refocusing was achieved by introducing a phase term into the pupil function during image reconstruction, a method we term here ‘initial pupil modification’ (IPM), the computational simplicity of post reconstruction refocusing (PRR) means it can be achieved in real time. We apply this method to analyze the 3D structure of Giemsa stained peripheral blood films and present an image processing workflow to yield a 2D extended depth of field (EDoF) image for diagnostic evaluation. Finally, by enabling numerical correction of chromatic focus shifts, we demonstrate how PRR can be applied to improve the quality of color FPM images.

## 2. Methods

### 2.1 FPM principle and hardware

**Fig. 1.**
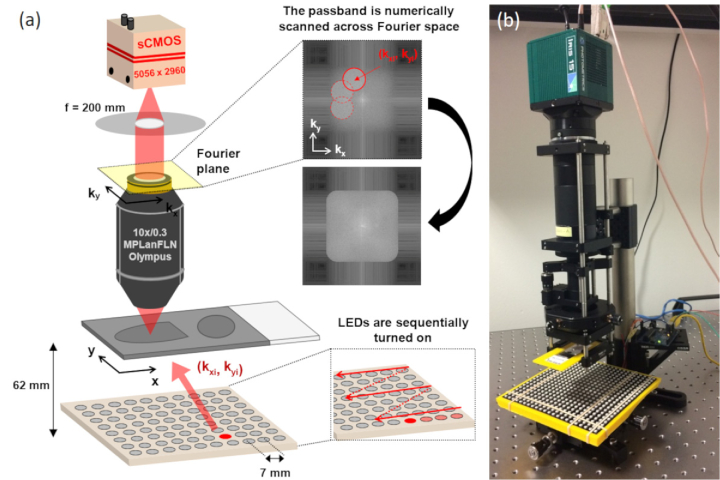
(a) Schematic diagram illustrating the principle of FPM in which images of the sample are captured at different illumination angles using an LED array. (b) Experimental FPM setup using a low-cost off-the-shelf LED matrix.

When a thin sample of transmittance *t*(*x,y*) is illuminated by a monochromatic plane wave *e_i_*(*x,y*) = exp[*ik*(*α_i_x*+*β_i_y*)] the resulting exit wave from the sample is given by *e*(*x,y*) = *t*(*x,y*)*e_i_*(*x,y*), where *k*, (*x,y*) and (*α_i_,β_i_*) denote the wavenumber, the 2D spatial coordinates in the sample and the illumination angle respectively. After passing through the objective lens of a microscope the spectrum of the exit wave, *E*(*u,v*), is multiplied by the pupil function of the objective lens *P*(*u,v*), where (*u,v*) are the spatial frequencies in the *x* and *y* directions. The intensity image recorded by the camera is thus given by

(2) $$I_{i}={\left|\text{iFT}[T(u-\alpha_{i},v-\beta_{i})\cdot P(u,v)]\right|}^{2}$$

where *T*(*u,v*) = FT[*t*(*x,y*)] is the Fourier spectrum of the sample. In other words, the portion of the sample spectrum transmitted to the image plane is shifted by the wavevector of the illumination allowing capture of spatial frequencies beyond the limit imposed by the passband of the objective lens. Information contained in images acquired under different illumination angles can be combined through an iterative phase retrieval algorithm [14], in which the real space amplitude corresponding to each shifted passband is replaced with the square root of the corresponding measured image intensity (Fig. 1(a)). In this way the Fourier spectrum of the sample is filled out by enforcing the coherent transfer function (CTF) support in Fourier space and the measured image intensity in real space.

To capture FPM images we built a simple upright FPM system composed of an array of 22 × 22 addressable RGB LEDs (BTF-lighting) mounted 62 mm below the front focal plane of a 10x/0.3 objective lens (MPLanFLN, Olympus), a tube lens with a focal length of 200 mm (TTL200-A, Thorlabs Inc.) and a digital camera (Iris 15, Photometrics) with 5056 × 2960 4.25 µm pixels giving a FoV of 1.9 mm x 1.1 mm (Fig. 1(b)). All images were acquired using µ-manager software [15] with the switching of LEDs in the illumination array synchronized to the global exposure period of the camera’s rolling shutter using a microcontroller (Arduino Uno). Each high-resolution complex image was reconstructed, using the sequential Gauss-Newton algorithm described by Tian et al [16,17], from 225 low-resolution images captured under sequential illumination of the sample with the red LEDs (emission maximum ∼ 632 nm) arranged inside a filled circle of diameter 11.2 cm. Using an exposure time of 100 ms, the total acquisition time was ∼ 30 seconds per image set. The effective nominal synthetic NA for the reconstructed images was 0.88, this increase in NA means that the DoF of a reconstructed image is only ∼ 15% of that of a raw image.

### 2.2 Preparation of Giemsa stained blood films

Blood films were prepared at the College of Medicine, University of Ibadan, Nigeria according to World Health Organization malaria microscopy standard operating procedures MM-SOP-01 to 06b. Briefly, a 12 µL and a 2 µL droplet from a finger prick blood sample were deposited on different parts of a cleaned glass microscope slide. The larger droplet was then spread across a circular region of diameter 15 mm using a pipette tip to create a thick film and the smaller droplet was spread along the length of the slide using a second clean glass slide to form a thin film. Blood films were then fixed by running methanol over the slide. After drying, slides were incubated with Giemsa solution and left for 10 minutes before flushing away excess stain using buffered water. Slides were then air-dried a second time before imaging. Healthy red blood cells (RBCs) are biconcave with a thickness of approximately 2.5 µm and a diameter of  6-8 µm. A fraction of RBCs were infected with the *P. falciparum* malaria parasite (MP), which is characterized by a ring-shaped morphology consisting of a dark, densely stained chromatin spot surrounded by a fainter cytoplasmic ring. High quality, high resolution images are essential for reliable identification of MPs, as real-world clinical samples often contain many false positive ‘parasite-like’ artefacts which can be difficult to distinguish from real MPs. As each MP may be located at any axial position within the RBC membrane, effective detection requires examination of images spanning the entire cell thickness.

### 2.3 Digital refocusing of FPM images

**Fig. 2.**
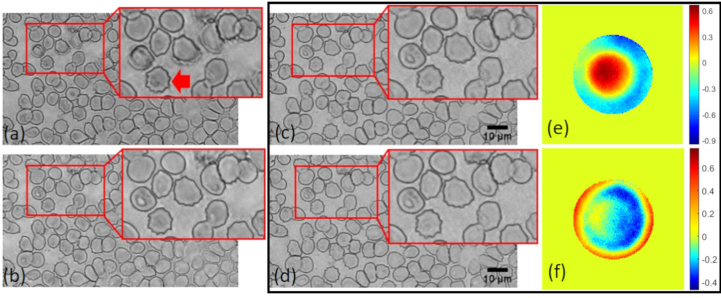
Observation of the decrease in the DoF after reconstruction. FPM amplitude images of a blood smear containing infected RBCs (red arrow) for raw images captured with the sample at two different axial positions within the DoF of the objective lens. (a)-(c) *z* = -2 µm; (b)-(d) *z* = 1 µm. (c) and (d) are aberration corrected. (e)-(f) Phase in radians of pupil functions corresponding to the system for cases (c) and (d).

We investigated digital refocusing using FPM images of thin blood films from malaria positive samples (in air) selected at random from our bank of blood film slides. In the first instance, each image was reconstructed assuming an ideal (diffraction limited) pupil function, leaving any imaging aberrations uncorrected. Figure 2(a-b) shows two reconstructed amplitude images with the sample at two different axial positions, Δ*z* = -2 µm and Δ*z* = 1 µm, within the DoF of the objective lens. The shallow DoF of the reconstructed images means that all features within the ∼ 3 µm thickness of the blood film are not simultaneously in focus. Specifically, we observe that MPs are clearly visible within the highlighted cell in (a) while they are barely noticeable in (b). On the other hand, the RBC membranes are optimally focused (most sharp) in (b), but out of focus in (a). The structural similarity index (SSIM) [18] of these two images was found to be 0.49, further emphasizing the difference between images reconstructed from raw data captured with the sample within the nominal DoF of the objective. To correct the optical aberrations of the system the same reconstructions were performed again by updating the pupil function in the recovery procedure using the embedded pupil function recovery (EPRY) algorithm [16,19] (Fig. 2(c-d)). The phase of the associated recovered pupil functions (e-f) contain significant defocus terms (Zernike coefficient $Z_{2}^{0}$ = -0.5 and 0.26 for cases (e) and (f) respectively). This further demonstrates a decrease in the DoF with effective NA increase in the reconstructed image. Finally we note the close similarity of the aberration corrected images reconstructed in Fig. 2(c-d) (SSIM = 0.71). This illustrates a general feature we observed for images reconstructed using the EPRY approach in that they are focused at a plane with maximal image contrast, in this case the plane at which the RBC membranes are sharpest consequently preventing the further detection of MPs that might be in a different plane.

As the FPM recovery procedure yields a complex image containing both amplitude and phase information, it is possible to use numerical propagation methods to estimate the field distribution at a different axial location and bring out of focus sample features into focus. When an optical field is known in a given plane *z*_0_, the field in a different parallel plane *z* can be calculated using the angular spectrum (AS) method in which the field is decomposed into a set of plane waves, each of which is then phase delayed to account for propagation [20]. Mathematically this is simply achieved by multiplying the Fourier transform (FT) of the field by a term *H*(*u,v*) that characterizes the propagation in the frequency domain and then performing an inverse Fourier transform (iFT) on the result (Eq. 3).

(3) $$\begin{array}{l} U(x,y,z)=\text{iFT}(\text{FT}[U(x,y,z_{0})]\cdot H(u,v))\;\\ \text{with }H(u,v)=\exp\left(j\frac{2\pi }{\lambda }z\sqrt{1-{\left(\lambda u\right)}^{2}-{\left(\lambda v\right)}^{2}}\right) \end{array}$$

Interestingly, we found that applying a different free space propagation term *H*(*u,v*) (Eq. (4)), which corresponds to a linear increase in phase delay with spatial frequency, in some cases leads to higher contrast in refocused images.

(4) $$H(u,v)=\exp\left(j\frac{2\pi }{\lambda }z\right)\exp\left(-j\frac{\pi }{\lambda }z\sqrt{{\left(\lambda u\right)}^{2}+{\left(\lambda v\right)}^{2}}\right)$$

Compared to the AS free space propagation kernel this linear kernel imparts a larger phase difference between plane waves propagating at low to intermediate angles and a smaller difference for large angle plane waves. This may increase the contrast at the low to intermediate frequencies where the signal to noise is highest, allowing prominent structures within the image to be more clearly visible.

**Fig. 3.**
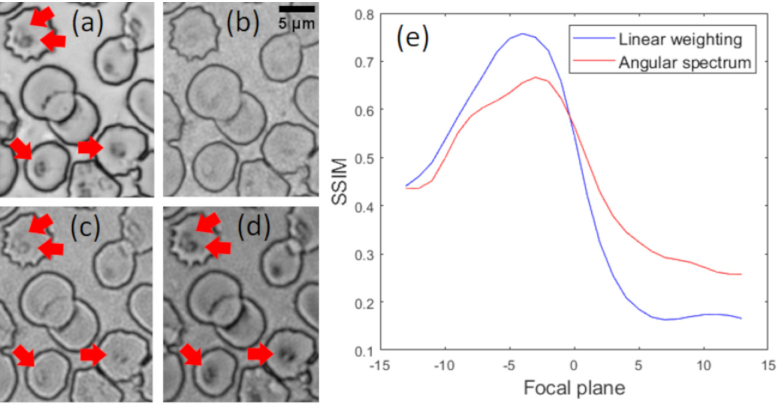
(a) Reference image captured from a 100x/0.8 objective manually focused at the plane for which MPs (indicated by red arrows) are optimally in focus. (b) FPM amplitude image generated using the autofocus EPRY method. (c-d) Optimal (highest SSIM) refocused images, using (c) the AS propagation term and (d) a linear weighting of frequency components. (e) SSIM of the different focal planes for both propagation kernels. Image (a) is used as the reference.

Both methods were experimentally tested by using a reconstructed FPM image of a Giemsa stained blood film containing three infected RBCs (Fig. 3(b)). To assess numerically refocused image results, a reference image of the same region highlighting the MPs (indicated by red arrows, Fig. 3(a)) was also obtained using an air objective (LMPlanFL 100x/0.8, Olympus) with an NA close to that synthetized by our FPM setup. To reduce spatial coherence artefacts the reference image was captured under illumination of the sample by a square patch of 5 × 5 LEDs. Figure 3(e) shows SSIM values (omitting the luminance term) computed against the reference for images digitally refocused using both AS and linear propagation kernels. The refocused images of Fig. 3(b) with the highest measured SSIM values (0.67 and 0.76 for AS and linear weighting methods respectively), are shown in Fig. 3(c) and (d) respectively. In addition to a higher peak SSIM value, visually the linear propagation kernel yields a refocused image which more closely resembles the raw high-resolution optical image (Fig. 3(a)).

### 2.4 Extended depth of field rendering of FPM images

**Fig. 4.**
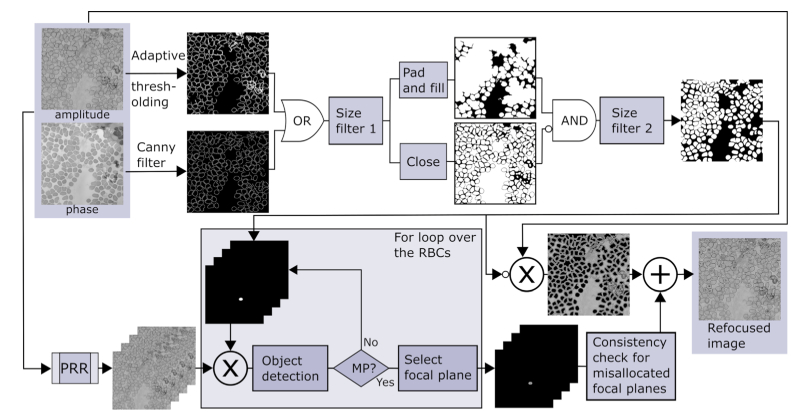
(a) Flow chart depicting the pipeline developed for generating an EDoF image from a reconstructed FPM image. Size filter 1 and size filter 2 refer to size exclusion filters to remove very small objects (less than fifty pixels) and objects smaller than a cell with a diameter of 4 µm respectively.

An important problem in 3D imaging is how to optimally represent and display 3D image information to facilitate analysis. This is particularly relevant in digital pathology where clinical diagnosis is based on analysis of image features by a trained expert or, increasingly, by a computer. In order to render a 2D image which effectively displays 3D image information (specifically both RBC membranes and MPs) we developed an image processing pipeline using focal stacks numerically generated using the PRR method. The depth of the stack was set to be greater than the entire thickness of the sample with a focus step size less than the synthetized DoF. For each focal plane two binary masks were created by applying a gradient-based edge detector to the unwrapped FPM phase image [21] and adaptive intensity thresholding to the amplitude image. An RBC segmentation mask was then created by combining these two masks through a logical OR operation, followed by removal of small objects and noisy pixels using a size exclusion filter and morphological filling. To deal with border effects, the mask was first padded by reflection before RBC filling. To separate clumped RBCs into individual objects a second mask was created using the same steps as previously but using morphological closing rather than filling. A final binary mask was then generated by combining the first mask with the complement of the second mask through a logical AND operation and excluding objects with an area of less than 12 µm^2^. This area threshold corresponds to approximately 50% of the expected area of a typical RBC. Candidate MPs within subregions of the focal series, each corresponding to an RBC in the binary image mask, were then identified by first performing automatic global thresholding within the cell membrane and then filtering out objects significantly smaller than the lower bound of the expected size of an MP (approximately 1 µm in diameter). Where a candidate MP was detected, the intracellular region of interest was replaced by the refocused plane for which the length of the object perimeter (determined using a Canny filter) was the closest to the perimeter of a typical MP (4 µm). Finally, a consistency check was performed to deal with any misallocated intracellular focal planes caused by debris or staining artefacts. A flow chart describing step-by-step our approach is shown in Fig. 4.

**Fig. 5.**
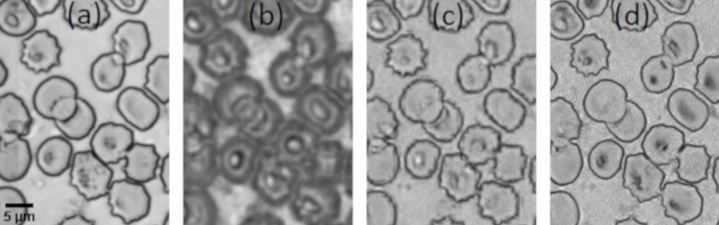
(a) Reference image captured using a 100x/0.8 objective lens (b-d) EDoF images computed using (b) the variance method (c) the complex wavelet transform method (d) our RBC segmentation method. The same numerically computed focal series was used for (b), (c) and (d).

We compared the results obtained using this method against conventional EDoF approaches [22–24] (Fig. 5). Visually our EDoF approach generates images significantly closer to the reference brightfield image. We attribute this to the presence of high spatial frequency reconstruction artefacts typically present in FPM images, which are problematic for conventional EDoF algorithms.

## 3. Results

### 3.1 Digital refocusing performance

**Fig. 6.**
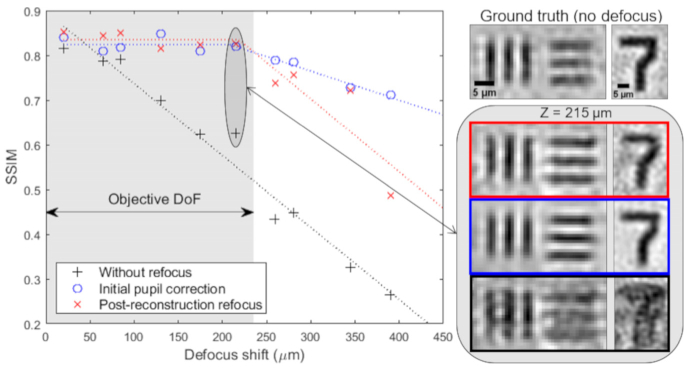
Performance of digital refocusing methods as a function of focus shift. The bottom right panel shows three reconstructed features without refocusing (black), using the IPM method (blue) and the PRR method (red) for a defocus distance of 215 µm.

We explored the limits of PRR by imaging a 3D test sample fabricated using two chrome on glass calibration targets separated by microscope cover glasses with a 1.25x/0.04 objective lens (PlanApo N, Olympus) with a nominal DoF (Eq. 1) of ∼ 470 µm at 632 nm. Raw images were acquired with the sample offset by various distances from the front focal plane of the objective lens using a micrometer driven translation stage. We then applied digital refocusing using both IPM and PRR methods and compared the results for four specific sample features (a 65 µm diameter disc, vertical and horizontal line pairs and the numeral ‘7’) to corresponding FPM reference images captured with the two calibration targets in focus. The results (Fig. 6) indicate that both digital refocusing methods allow similarly accurate recovery of a focused image provided the object is within the DoF of the objective lens. However, the quality of the refocused images starts decreasing as the object is moved outside the nominal DoF of the objective lens.

Compared to IPM, PRR is substantially less computationally intensive. In the IPM method, image reconstruction is repeated for each refocus step by modifying the EPRY recovered pupil function to include an additional defocus term. Despite initial knowledge of the system aberrations, we found that typically ∼150 iterations of the phase retrieval algorithm were required for convergence to a good quality reconstructed image, which was assessed by measuring the SSIM value for the refocused image against the image reconstructed using the EPRY algorithm. To measure the difference in processing time we reconstructed a high resolution, aberration corrected FPM image using the EPRY method and then used both PRR and IPM refocusing methods to generate focal series. Using a standard desktop PC equipped with Intel(R) Xeon(R) CPU E5-1650 processor at 3.20 GHz 16 GB RAM, we measured average per plane refocusing times of 55 ms for the PRR and 185 seconds for the IPM methods for an 600 × 600 pixel image. For the case of a thin blood film imaged with our FPM setup (Fig. 1(b)) we typically require a minimum of twelve focal planes to capture the entire thickness of the sample, which takes < 1 second to compute using the PRR method compared to ∼ 37 minutes using the IPM method.

### 3.2 Extended depth of field FPM imaging of blood films

**Fig. 7.**
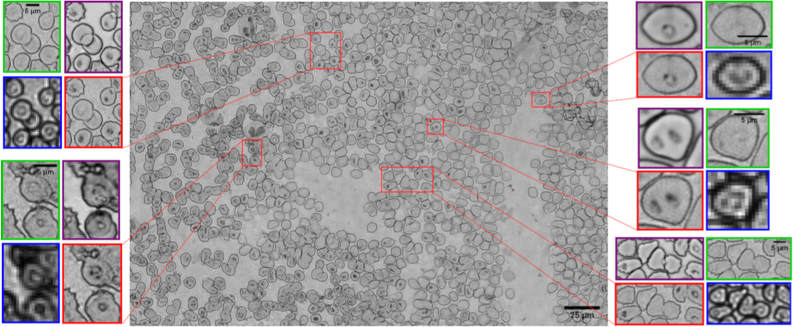
EDoF image of a Giemsa stained blood film captured in air. For each highlighted boxed region (red) the corresponding raw captured image (blue), the reconstructed (unfocused) high resolution image (green) and a reference image captured using a 100x/0.8 objective lens (purple) are also shown.

Figure 7 shows a large reconstructed EDoF image of a blood film formed by independently processing and stitching together raw image data for six smaller image patches. Insets show comparison of the EDoF images (red squares) against raw images captured using a 100x/0.8 objective lens (purple squares), demonstrating the ability of the EDoF method to render intracellular features which are out-of-focus in the initial reconstructed image (green squares), whilst retaining sharp, focused RBC membranes. Requiring only two fast Fourier transform operations, a series of matrix multiplications and basic image processing operations, the full EDoF reconstruction method took only 25 seconds to process a focal series with 21 planes on a modest specification PC (as previously), even for a dense sample containing ∼ 250 RBCs. Although the EDoF reconstruction time depends upon the number of detected RBCs, each RBC is analyzed independently and so processing time could be substantially reduced by parallel processing using a multi-core CPU or GPU.

### 3.3 Correction of chromatic focus shifts using digital refocusing

**Fig. 8.**
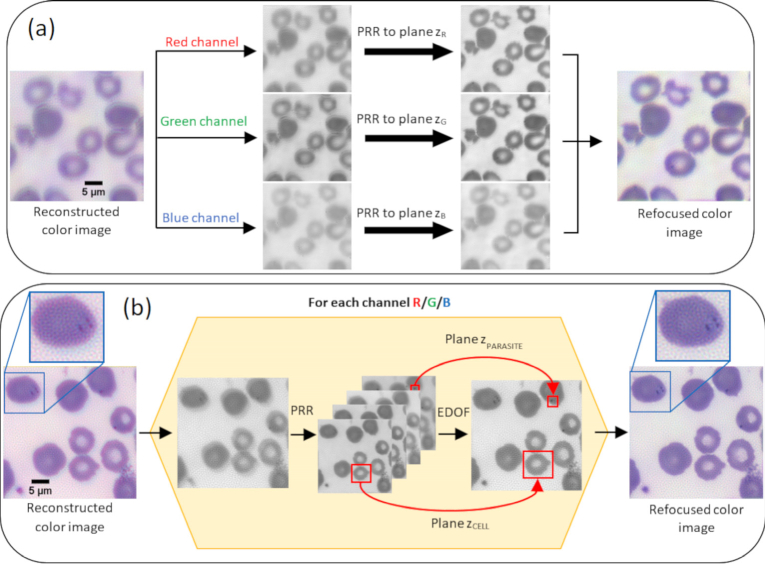
Application of PRR and EDoF methods to correct chromatic focus shifts in FPM. (a) (left) A blood smear sample coated with oil is located outside the DoF of the objective lens for two of the three color channels. (centre) Individual color channel images can be refocused using PRR (where z_R_, z_G_, z_B_ are different planes within the numerically generated focal series), before being combined into a final corrected RGB image (right). (b) (left) Reconstructed FPM image of the same sample positioned within the DoF of the objective lens for all illumination wavelengths. (centre) 2D EDoF images are reconstructed separately for each color channel, to optimally display 3D image information. These single color EDoF images can be combined to yield a final color EDoF image free from artefacts associated with chromatic focal shifts (right).

In FPM, color images are typically generated by combining independent image datasets captured under illumination of the sample with red, green and blue LEDs. However, the low magnification, low NA objective lenses generally employed in FPM setups often suffer from significant chromatic focus shifts resulting in poor quality images when the color channels are combined. By independently refocusing the color channels before combining them, the PRR and EDoF approach can also be applied to correct these offsets and render a significantly improved color FPM image. Figure 8(a) illustrates the effectiveness of digital refocusing for correcting chromatic focus shifts using the example of an oil coated blood film. In this case the blood film was positioned outside the DoF of the objective lens for the green and red color channels. We used PRR to generate a focal series for each color channel independently. The axial offset between these three focal series was then measured by determining the difference in position of the sharpest plane within each series (z_R_, z_G_, z_B_), where the sharpest image plane was determined by first applying a Sobel edge detection operator to the median filtered images. Of the two images with the highest intensity (strongest edges) the sharpest plane was then defined as the image with the highest variance. After applying a corresponding axial offset, color channels were then merged to create a significantly improved RGB color image. However, the fact that two of the three color images were captured outside the DoF of the objective lens precludes recovery of an optimally sharp color image (as discussed in section 3.1). Figure 8(b) illustrates how the PRR EDoF method can be extended to allow rendering of in focus color images. The panel on the left shows a reconstructed FPM image of the same blood film sample as in Fig. 8(a), this time axially shifted such that it lies within the DoF of the objective lens for all illumination wavelengths. Due to the reduced DoF, chromatic focus shifts degrade the quality of the reconstructed color image, resulting in changes to the hue of the parasites (which appear redder) and a blue halo around the edge of the RBCs. To generate the color EDoF image shown on the right-hand side of the figure we slightly modified the EDoF image processing pipeline described in 2.4 to account for the change in appearance of the RBCs in immersion oil compared to air. The partial refractive index matching of the sample by the immersion oil means RBCs are no longer surrounded by a high contrast dark edge (as in Fig. 7) and the contrast of the cytoplasmic ring in each MP is reduced. In this case RBC segmentation masks were computed by first applying a logical OR operation to binarized images of the amplitude and phase. To facilitate separation of clumped RBCs, a second binary image was generated by applying a Sobel edge detector to the amplitude and phase images and then combining them through a logical OR operation. As previously, a final RBC segmentation mask was then computed by combining these two binary images through a logical AND operation before performing filling, padding and filtering operations. To generate the final EDoF image, the image was first set to plane where the RBC membranes are optimally focused (as explained above) and then the intracellular region of each RBC was replaced by the digitally refocused plane at which the contrast of the detected MP was maximized. Finally, fusing individual EDoF images of red, green and blue color channels results in a color EDoF image free from the artefacts induced by chromatic focus shifts.

## 4. Conclusion & discussion

We have shown how digital refocusing by numerical propagation of the complex field recovered in FPM allows effective visualization of the structure of samples of intermediate thickness, facilitating further analysis. In particular, we found that PRR yields refocused images comparable in quality to those obtained by refocusing the FPM dataset through modification of the pupil function within the iterative phase retrieval algorithm, however requires a small fraction of the computational effort. This makes it feasible to generate large focal series, permitting fast digital refocusing of images without prior knowledge of the focus offset and also for visualizing FPM images of curved or tilted substrates. We also showed how PRR allows effective correction of the chromatic focus shifts which are often a feature of the low magnification, low NA lenses generally used in FPM systems. Further, we demonstrated how a digitally generated focal series of a 3D sample can be processed to generate an EDoF image and applied this approach to allow simultaneous visualization of RBC membranes and MPs in a Giemsa stained blood film. Given both, its computational efficiency and speed, this method offers significant potential to extend the application of FPM to visualize and analyze a broad range of 3D samples.

Throughout we have adopted the assumption that samples are sufficiently thin that a tilt in illumination corresponds to a simple shift in the captured portion of the sample spectrum. Ou et al. discuss this assumption in detail in [7], deriving a theoretical thickness limit of *h*_max_ = **π**/|*k_z_*|_max_ based on consideration of the variation in the axial wavevector *k_z_* with illumination angle. For our FPM system this suggests a maximum thickness limit of 2.7**λ** or 1.7 µm at 632 nm, which is somewhat less than the expected thickness of the cells in a thin blood film. However, as Ou et al. point out, this limit applies to samples with a transmittance function which varies rapidly in the axial direction. For samples which are relatively uniform or slowly varying axially we expect the practical thickness limit to be significantly larger, suggesting these methods will be valid for visualization and analysis of many samples from cell monolayers to tissue sections. For samples which are substantially thicker, more computationally complex full 3D FPM reconstruction schemes, such as those based on multi-slice reconstruction [13] or diffractive tomography [10], are required.
